# Intracranial Pseudoaneurysms: Evaluation and Management

**DOI:** 10.3389/fneur.2020.00582

**Published:** 2020-07-07

**Authors:** Yongtao Zheng, Zheng Lu, Jianguo Shen, Feng Xu

**Affiliations:** ^1^Department of Neurosurgery, Huashan Hospital, Shanghai Medical School, Fudan University, Shanghai, China; ^2^Department of Neurosurgery, Hai'an People's Hospital, Nantong, China; ^3^Department of Neurosurgery, Second Affiliated Hospital of Jiaxiang University, Jiaxing, China; ^4^Department of Neurosurgery, Kashgar Prefecture Second People's Hospital, Kashgar, China

**Keywords:** intracranial pseudoaneurysms, trauma, iatrogenic, management, endovascular treatment

## Abstract

Intracranial pseudoaneurysms account for about 1% of intracranial aneurysms with a high mortality. The natural history of intracranial pseudoaneurysm is not well-understood, and its management remains controversial. This review provides an overview of the etiology, pathophysiology, clinical presentation, imaging, and management of intracranial pseudoaneurysms. Especially, this article emphasizes the factors that should be considered for the most appropriate management strategy based on the risks and benefits of each treatment option.

## Introduction

Intracranial pseudoaneurysm is a rare entity and represents about 1% of all intracranial aneurysms, with an associated mortality of 20% or higher (1). The most common cause of pseudoaneurysm is trauma (2). Other causes are iatrogenic, infectious disease, radiation exposure, connective tissue disease, and sometimes they occur spontaneously (3–6). A pseudoaneurysm or false aneurysm is the product of damaging vessel wall resulting in an encapsulated hematoma in communication with the ruptured artery. Clinical presentations may vary depending on the rupture status, location, and size of the intracranial pseudoaneurysm (7). If untreated, the mortality rate for patients with intracranial pseudoaneurysm can reach high up to 50% due to delayed rupture and disastrous bleeding (1, 8, 9). Therefore, early diagnosis and efficient treatment are mandatory. In this review, we provide a comprehensive evaluation of the risks and benefits of different treatment options available for pseudoaneurysms, such as observation, microsurgical clipping, and endovascular embolization. Besides, the etiology, pathophysiology, clinical presentation, and imaging of intracranial pseudoaneurysms are also discussed.

## Classification

Pseudoaneurysms account for about 1% of aneurysms in adults; however, the incidence rate in the pediatric group is more than 19% (9, 10). Anatomical anomalies, venous sinus thrombosis, multiple surgeries, and prior radiotherapy increase the incidence rate of the pseudoaneurysm. Intracranial pseudoaneurysms can be classified as traumatic, infectious, iatrogenic, and other types.

### Traumatic Pseudoaneurysms

Head trauma is the most common cause of intracranial pseudoaneurysms. Closed or penetrating head trauma to cerebral blood vessels, which lead to pseudoaneurysm, could be classified as direct or indirect (1). A penetrating wound resulting from a variety of weapons and cutlery leads to direct vascular injury. Indirect vascular trauma is often encountered in seriously closed brain injuries, such as traffic accidents and bony prominences during major brain shifts.

### Infectious Pseudoaneurysms

Infectious pseudoaneurysms can be caused by bacteria, tuberculous bacilli, or fungi (11–13). In comparison with other intracranial aneurysms, infectious aneurysms have a slight preference for younger people. Ruptured aneurysms have a higher rate of mortality. Most of the infectious pseudoaneurysms are located in the anterior circulation, and those aneurysms can be multiple in many cases. The definition of infectious pseudoaneurysms should be based on the angiographic features and demonstration of infection.

### Iatrogenic Pseudoaneurysms

In addition to trauma and infection, iatrogenic vascular injury is another important cause of intracranial pseudoaneurysms. They generally involve the internal carotid artery (ICA) due to ICA injury after transsphenoidal or transcranial resection of sellar region tumors (6, 14–19). Iatrogenic pseudoaneurysm are less common in the anterior cerebral artery (12, 20–24), the basilar artery (18, 25–27), and the middle cerebral artery (22, 28–30). Pseudoaneurysms after mechanical thrombectomy or stent angioplasty is one of the potential complications associated with the endovascular procedure (31, 32). Although rare, we should raise the suspicion for this potentially lethal complication.

### Other Types of Pseudoaneurysms

Other causes of intracranial pseudoaneurysms include Marfan's syndrome, fibromuscular dysplasia, vasculitis, rupture of true cerebral aneurysm or arteriovenous malformation, associated with moyamoya disease, and radiotherapy (4, 5, 33, 34). Dissecting pseudoaneurysms and blood blister-like aneurysms were out of the scope of the discussion.

## Pathophysiology

Compared with the extracranial arteries, the intracranial arteries are thinner and stiffer. They have a thinner media and adventitia, absence of an external elastic lamina, and possess a thicker internal elastic lamina (35, 36). These features make the intracranial arteries more vulnerable to trauma.

**Figure 1:**
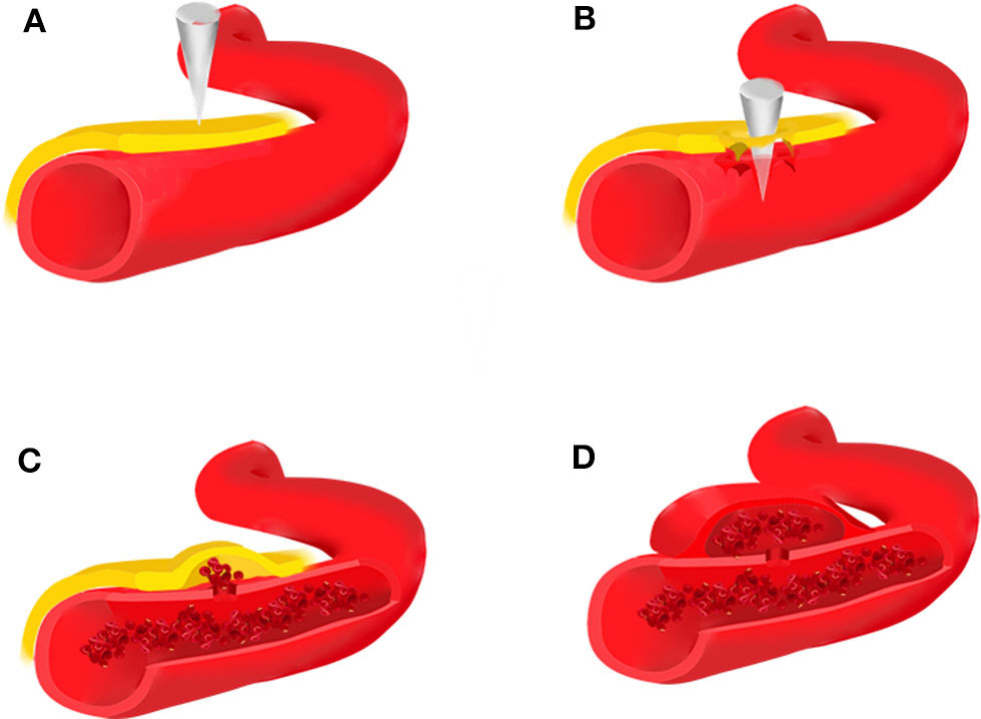
Brain trauma leads to rupture of the intima, media, and adventitia of the blood vessel **(A,B)**, forming an organized hematoma cavity **(C)**. When the hematoma forms outside the arterial wall, it continues to communicate with the injured vessel, thus predisposing it to re-bleeding **(D)**.

Traumatic intracranial aneurysms can be histologically categorized as true or false. True aneurysms usually develop following a partial disruption of the arterial wall. The intima, internal elastic lamina, and media are damaged, whereas the adventitia is intact (1, 3, 8, 36, 37). False aneurysms or pseudoaneurysms result from disruption of the entire arterial wall (Figure 1). A contained hematoma forms outside the vessel, being restricted by perivascular connective tissues. However, it continues to communicate with the injured artery and is more likely to rebleed.

Iatrogenic pseudoaneurysms can be classified as saccular and fusiform (36). Saccular pseudoaneurysms occur secondary to penetration or complete laceration of the arterial wall. They lack a true wall and are only contained by a fragile layer of connective tissue (7, 19). Fusiform pseudoaneurysm may result from thinning of the adventitia during surgical peeling of tumor from the adjacent vessel. In comparison to saccular pseudoaneurysms, they usually do not rupture.

A pseudoaneurysm can also form at the tip of the “true” aneurysm (34, 38). We speculated that it might be the thick hematoma around the aneurysm (38). The temporary disorder of cerebrospinal fluid circulation may also play a significant role in the formation of pseudoaneurysm, since the blood extravasating from the vessel could gather into a hematoma but not diffuse into the cerebrospinal fluid. Thus, a sufficient volume of the subarachnoid pool may result in the occurrence of the pseudoaneurysm (34).

## Clinical Presentation

Intracranial pseudoaneurysms may present with intracranial hemorrhage, epistaxis, headaches, seizures, neurological deficits, and associated with other cerebrovascular diseases.

Intracranial hemorrhage is the most common presentation, manifesting as acute hemorrhage associated with the initial injury or in a delayed manner (39). Intraoperative arterial bleeding occurred in the majority of patients with iatrogenic vascular injury. Patients with no evidence of vascular injury during the operation may suffer postoperative or delayed hemorrhage, including intracerebral, intraventricular, and subarachnoid hemorrhage. Pseudoaneurysms of the middle meningeal artery typically are associated with epidural or subdural hematoma (40, 41).

Epistaxis is the common symptom of intracavernous ICA pseudoaneurysms. The intracavernous ICA is close to the sphenoid sinus, mostly bulging into the lateral sinus wall (37). A congenitally thin or even absent bony structure covering the cavernous ICA within the sphenoid sinus may provide less protection against bony erosion (42). Massive epistaxis may be caused by erosion of the lateral wall of the sphenoid sinus. We also cannot exclude the internal maxillary artery as another source of hemorrhage (9). Epistaxis may be delayed in 7 days to 8 months after trauma or iatrogenic intracavernous ICA injury. The initial episodes of epistaxis may be mild and not fatal. However, recurrent bleeding can lead to fatal blood loss. Thus, we should not neglect it, in order to prevent delayed diagnosis and treatment.

Focal neurological deficits are often associated with traumatic pseudoaneurysms of the ICA. Due to its proximity to other cavernous structures, including cranial nerves II, III, IV, V1, V2, and VI. Traumatic ICA pseudoaneurysms may present with cranial nerve deficits, unilateral blindness, or a carotid-cavernous fistula associated to skull base fractures (9, 11, 37, 43). Other symptoms include headache, seizures, neck rigidity, decreased mental state, paralysis, or reduced level of consciousness.

## Imaging

Due to the high mortality related to pseudoaneurysm rupture, early diagnosis is mandatory. If intraoperative arterial bleeding occurs, iatrogenic vascular injury should be suspected. Angiography should be used for patients with perioperative hemorrhage or epistaxis. Digital subtraction angiography (DSA) is still the gold standard for the diagnosis of intracranial pseudoaneurysms since CTA and MRA have limited sensitivity for the detection of small aneurysms (44) DSA often demonstrates a globular shaped aneurysmal sac without a neck (5). Delayed filling and stagnation of contrast agents are the features of the pseudoaneurysm. If the initial imaging is negative, angiography shoud be repeated because pseudoaneurysms often develop days to weeks after injury. The optimal time interval between angiographies is still a matter of debate. Some studies reported negative initial angiograms within several hours or days of trauma. Follow-up angiograms showed an aneurysm weeks to months later. Therefore, initial angiography was suggested to be performed 1 or 2 weeks after vascular injury to avoid missed diagnoses (39, 44). However, another study recommends it 6 and 12 months postoperatively (15).

## Management

As the causes of pseudoaneurysms are different, treatment options are challenging. Management of intracranial pseudoaneurysms includes microsurgery, embolization, and conservative treatment (Tables 1, 2).

**Table 1:** Literature review of intracranial pseudoaneurysms treated with microsurgery^*^.

**References**	**Age (ys) /Sex**	**Artery involved**	**Etiology**	**Presentation**	**Treatment**	**Immediate aneurysm occlusion**	**Procedure-related complication**	**Outcome**	**Follow-up angiogram**
Akamatsu et al. (4)	75/F	Distal AICA	Radiation	SAH	Trapping and resection	Complete	NA	NA	NA
Binning et al. (45)	16ws/F	ICA C7	Traumatic	SAH	Surgical suturing /wrapping-clipping	Complete	None	Good	NA
Chen et al. (22)	25/F	MCA branch	Iatrogenic	Delayed ICH	Direct clipping	Complete	None	Good	No recurrence
Cikla et al. (36)	68/M	ICA C6	Iatrogenic	Intraoperative bleeding	Trapping and bypass	Complete	None	Good	NA
Ding et al. (3)	37/M	ACoA	Unknown	SAH	A fenestrated clip	NA	Intraoperative rupture	Good	NA
	50/F	ICA C7	Unknown	SAH	An encircling clip	NA	Aneurysm avulsion	mRS 3	NA
Horiuchi et al. (8)	66/M	Distal MCA	Traumatic	Delayed ICH	Trapping and resection	Complete	None	mRS 2	NA
Imahori et al. (32)	84/F	MCA M2	Iatrogenic	Delayed ICH	Surgical suturing	Complete	None	mRS 4	NA
Kumar et al. (39)	49/M	Frontopolar	Traumatic	ICH	Direct clipping	Complete	None	mRS 4	NA
	20/F	ACA A3/A4	Traumatic	ICH	Direct clipping	Complete	None	Good	NA
Le et al. (20)	30/M	ACA A4	Iatrogenic	Delayed ICH	Resection	Complete	None	NA	NA
Raper et al. (46)	71/F	AChA	Unknown	SAH	Surgical trapping	Complete	None	Good	NA
Ravina et al. (23)	43/M	Proximal A3	Iatrogenic	SAH/ICH/nfarcts	Trapping and bypass	Complete	None	mRS 6	/
	20/M	Proximal A3	Traumatic	Recurrent ICH/SAH	Trapping and bypass	Complete	None	mRS 5	NA
	11/F	A1-A2	Iatrogenic	Epistaxis/SAH	Trapping and bypass	Complete	None	mRS 4	NA
Rayes (28)	22/M	MCA M4	Iatrogenic	Postoperative ICH	Resection and end-to-end anastomosis	Complete	None	Good	NA
Sato et al. (47)	61/F	Distal LSA	Unknown	IVH	Resection	Complete	None	Good	NA
Shirane et al. (48)	40ws/F	ICA C7	Iatrogenic	Postoperative IVH	Surgical suturing	Compete	None	Good	No recurrence
Sujijantarat et al. (49)	14/M	BA	Traumatic	Extensive SAH	Staged trapping	Complete	None	mRS 3	NA
Umekawa et al. (50)	78/M	Distal AICA	Radiation	VII/VIII palsy	Trapping and bypass	Complete	None	mRS 2	NA
Walcott et al. (51)	26/M	ACA A2	Traumatic	CTA discovered	Trapping and bypass	Complete	None	Good	No recurrence

* *Pseudoaneurysms of the middle meningeal artery were not included*.

*ACA, anterior cerebral artery; AChA, anterior choroidal artery; ACoA, anterior communicating artery; AICA, anterior inferior cerebellar artery; BA, basilar artery; CTA, computed tomography angiography; ICA, internal carotid artery; ICH, intracerebral hemorrhage; IVH, intraventricular hemorrhage; LSA, lenticulostriate artery; MCA, middle cerebral artery; mRS, modified Rankin Scale; NA, not available; SAH, subarachnoid hemorrhage; ws, weeks*.

**Table 2:** Literature review of intracranial pseudoaneurysms treated with endovascular embolization from 2010^*^.

**References**	**Age (ys) /Sex**	**Artery involved**	**Etiology**	**Presentation**	**Treatment**	**Immediate aneurysm occlusion**	**Complication**	Outcome^*^	**Follow-up angiogram**
Al-Jehani et al. (52)	28/M	Cavernous ICA	Traumatic	Epistaxis	Coiling	Near complete	None	Good	No recurrence
Aljuboori et al. (53)	19/M	M2/ICA C6	Traumatic	SAH	1st coiling; 2nd flow diversion	Complete	None	Good (mRS 1)	No recurrence
Altali et al. (9)	6/M	Intracavernous ICA	Traumatic	Epistaxis/otorrhagia	Coiling	Complete	None	mRS 2	Recurrence; retreatment
Amenta et al. (7)	64/F	ICA C5	Iatrogenic	Intraoperative bleeding	Flow diversion×2	Decreased filling	None	Good	Complete
Chen et al. (22)	39/M	Distal call. marg.	Iatrogenic	Recurrent ICH	Aneurysm occlusion and PAO (Glubran)	Complete	None	Good	NA
Colby et al. (30)	9ms/F	MCA M1	Iatrogenic	Delayed ICH	1st coiling; 2nd flow diversion + coiling	Incomplete	None	Good	Complete
Fu et al. (11)	58/M	Cavernous ICA	Infectious	Epistaxis	1st coiling; 2nd trapping	Complete	Infarction	mRS 4	NA
Giorgianni et al. (54)	20/M	Right A1; left A2	Traumatic	ICH	Flow diversion×2	Complete; Complete	None	Good	No recurrence
Giorgianni et al. (55)	66/M	Intracavernous ICA	Iatrogenic	Intraoperative bleeding	Flow diversion	Complete	None	Good	No recurrence
Griauzde et al. (56)	7/F	BA	Traumatic	MRI discovered	Stent-assisted coiling	Near complete	None	NA	No recurrence
Griauzde et al. (18)	18/F	BA	Iatrogenic	Intraoperative bleeding	1st stent-assisted coiling; 2nd flow diversion + coiling	Complete	None	Good (mRS 0)	No recurrence
	49/F	Cavernous ICA	Iatrogenic	Intraoperative bleeding	Flow diversion×2	Complete	None	Good (mRS 0)	No recurrence
	60/M	ICA	Iatrogenic	Intraoperative bleeding	Flow diversion	Complete	None	Good (mRS 1)	No recurrence
Hjortoe et al. (16)	44/M	Cavernous ICA	Iatrogenic	Intraoperative bleeding	Coiling	Complete	None	Good	Recurrence
	63/F	OA	Iatrogenic	Delayed ICH	Coiling	Complete	None	NA	Recurrence
Jadhav et al. (12)	74	ACA A3	Iatrogenic	Postoperative SAH	Trapping (Onyx-34)	Complete	None	NA	NA
	61	Distal MCA	Mycotic	Aortic endocarditis	Trapping (Onyx-34)	Complete	Perforation	Good	NA
	38	ACA A2	Iatrogenic	Postoperative SAH	Trapping (Onyx-34)	Complete	None	NA	No recurrence
	56	ACA A2	Iatrogenic	Postoperative SAH	Trapping (Onyx-34)	Complete	Thrombosis	mRS 3	NA
	30	MCA	Mycotic	Infective endocarditis	Trapping (Onyx-34)	Complete	None	NA	No recurrence
Kim et al. (57)	13/F	ACA A2	Traumatic	SDH	Stent-assisted coiling	Complete	None	mRS 2	No recurrence
Kumar et al. (39)	47/F	ACA A3	Traumatic	Delayed SAH	Trapping (coils)	Complete	None	Death	/
Lee and Luo(24)	37/M	BA	Iatrogenic	Epistaxis	Coiling	Complete	Rebleeding	Death	/
Lim et al. (31)	60/F	MCA M1	Iatrogenic	Intraoperative bleeding	Overlapping stents	Near complete	Thrombosis	Good	Complete
Lim et al. (58)	30/M	Supraclinoid ICA	Traumatic	SAH	Stent-assisted coiling + a stent-within-a-stent	Complete	None	Good	No recurrence
Liu et al. (59)	15/F	ICA C6/C7	Traumatic	Epistaxis	Covered stent×2	Decreased filling	None	Good	Complete
	15/M	ICA C7	Traumatic	Headache	Covered stent	Decreased filling	None	Good	Complete
Liu et al. (60)	49/M	ACA A1	Traumatic	Epistaxis	1st coiling; 2nd PAO (coils+Onyx-18)	Complete	None	Good	NA
Mascitelli et al. (61)	65/M	Distal AICA	Radiation	SAH	PAO (nBCA)	Complete	Infarction	mRS 2	No recurrence
Matsumura et al. (62)	64/F	Distal AICA	Radiation	SAH	PAO (coils)	Complete	None	mRS 4	NA
	43/F	Distal ACA/PICA	Radiation	SAH	PAO (coils)	Complete	None	mRS 0	NA
Morinaga et al. (63)	68/M	PCoA	Iatrogenic	Recurrent SAH	1^st^ coiling; 2^nd^ LVIS stent-assisted coiling	Complete	None	Good	No recurrence
Munich et al. (24)	60	Frontopolar	Iatrogenic	ICH/SAH	PAO (coils+Onyx-34)	Complete	None	Aphasia	NA
Murakami et al. (64)	61/M	AICA (pontine)	Radiation	SAH	PAO (coils)	Complete	Infarction	mRS 2	No recurrence
Nariai et al. (19)	62/M	Cavernous ICA	Iatrogenic	Epistaxis	Flow diversion	Near complete	None	Good	Complete
Ogilvy et al. (65)	4/M	ICA C6	Iatrogenic	MRI discovered	Stent-assisted coiling	Near complete	None	Good	Complete
OuYang et al. (13)	49/M	Cavernous ICA	Infectious	Epistaxis	Stent-assisted coiling	Complete	Rebleeding	Death	/
Patel et al. (66)	56/M	Cavernous ICA	Iatrogenic	Intraoperative bleeding	1st, 2nd: Balloon-assisted (Onyx-500)	1st: Near complete; 2nd: Complete	None	Good	No recurrence
Sami et al. (67)	NA	Cavernous ICA	Iatrogenic	NA	Flow diversion×3	Decreased filling	None	Good (mRS 0)	Near complete
	NA	Cavernous ICA	Iatrogenic	NA	Flow diversion	Decreased filling	None	Good (mRS 0)	Complete
	NA	ICA C6	Traumatic	NA	Flow diversion×2	Decreased filling	None	Good (mRS 1)	Complete
	NA	PCA P1	Traumatic	NA	Flow diversion	Decreased filling	None	Good (mRS 0)	Complete
	NA	Cavernous ICA	Traumatic	NA	Flow diversion×2	Decreased filling	None	mRS 3	Complete
	NA	Cavernous ICA	Traumatic	NA	Flow diversion	Incomplete	Perforation	Death	/
	NA	ACA A3	Traumatic	NA	Flow diversion	Decreased filling	None	Death	/
	NA	Cavernous ICA	Traumatic	NA	Flow diversion	Decreased filling	None	Good (mRS 0)	Complete
Sastry et al. (26)	13/M	BA	Iatrogenic	IVH	1st coiling; 2nd coiling + flow diversion	Complete	None	Good	No recurrence
Shah et al. (29)	27/M	MCA M4	Iatrogenic	Delayed ICH	Trapping (nBCA)	Complete	None	mRS 3	NA
Van Rooij and Van Rooij (68)	28/M	Distal pericall. artery branch	Traumatic	ICH	Trapping (nBCA)	Complete	None	Recovered	NA
	22/M	Distal pericall. artery branch	Traumatic	Delayed ICH	Trapping (coils)	Complete	None	Recovered	NA
Wang et al. (2)	38/M	ICA C4	Traumatic	Eye blindness	Covered stent	Complete	None	Full recovery	No recurrence
	35/M	ICA C5	Traumatic	Epistaxis	Covered stent	Incomplete	None	Full recovery	Complete
	60/M	ICA C6	Traumatic	Headache/ptosis	Covered stent×2	Incomplete	None	Full recovery	Complete
	11/M	ICA C7	Traumatic	Decreased vision	Covered stent	Complete	None	Full recovery	No recurrence
	36/M	ICA C7	Traumatic	Decreased vision	Covered stent	Complete	None	Full recovery	No recurrence
	28/M	ICA C6	Traumatic	Epistaxis	Covered stent	Complete	None	Full recovery	No recurrence
	38/M	ICA C4	Traumatic	Epistaxis	Covered stent	Complete	None	Full recovery	No recurrence
	40/F	ICA C6	Traumatic	Decreased vision	Covered stent	Incomplete	None	Full recovery	No recurrence
	16/M	ICA C7	Traumatic	Decreased vision	Covered stent	Complete	None	Full recovery	No recurrence
	22/M	ICA C4	Traumatic	Epistaxis	Covered stent	Complete	None	Full recovery	No recurrence
	44/M	ICA C4	Traumatic	Eye blindness/ptosis	Covered stent	Complete	None	Improvement	No recurrence
	51/M	ICA C4	Traumatic	Epistaxis	Covered stent	Incomplete	None	Unchanged	Incomplete
Zanaty et al. (69)	55/M	ICA C7	Iatrogenic	Intraoperative bleeding	Flow diversion	Complete	None	Good	NA

* *Pseudoaneurysms of the middle meningeal artery were not included*.

*ACA, anterior cerebral artery; AChA, anterior choroidal artery; AICA, anterior inferior cerebellar artery; BA, basilar artery; Call. Marg., callosal marginal; CTA, computed tomography angiography; ICA, internal carotid artery; ICH, intracerebral hemorrhage; MCA, middle cerebral artery; MRI, magnetic resonance imaging; NA, not available; nBCA, n-butylcyanoacrylate; OA, ophthalmic artery; PAO, parent artery occlusion; PCA, posterior cerebral artery; PCoA, posterior communicating artery; Pericall., pericallosum artery; SAH, subarachnoid hemorrhage; SDH, subdural hemorrhage*.

### Microsurgery

Surgical intervention is typically reserved for the lesions of difficult catheterization or failed endovascular therapy, and presence of significant mass effect in ruptured pseudoaneurysms with acute hematoma, usually followed by clot evaculation and/or decompressive craniectomy. Direct surgery to treat pseudoaneurysms of the cavernous and petrous ICA is difficult. In the distal branch of intracranial arteries, such as the pericallosal artery, surgery still should be considered as an irreplaceable option. Surgical options include direct clipping, suturing, wrapping-clipping, ligation of the parent artery, and trapping with or without bypass (Table 1). However, different experts hold a variety of opinions on surgical strategies for pseudoaneurysms. Direct clipping of the aneurysmal neck may not be feasible because of the lack of a true vessel wall that makes clipping threatening and challenging (9, 45, 53). It often results in aneurysm avulsion and intraoperative bleeding due to the high fragility of the pseudowall (22). The orifice or defect can be repaired with direct microsurgical suturing (32, 45, 48). Subsequent wrapping-clipping supports the fragile wall and maintains the connectivity of the parent artery (45). Ligation of the parent artery can result in distal ischemic complications. Moreover, it may not prevent the rupture of pseudoaneurysms due to collateral retrograde flow into the lesion (37). Trapping, in which clips are placed on the parent vessel, both proximal and distal to the aneurysm, is the definitive treatment modality to eliminate the aneurysm. Trapping with or without bypass revascularization depends on the status of collateral supply. A low-flow bypass is often used to treat distal pseudoaneurysms (50), while a high-flow bypass is recommended for ICA pseudoaneurysms (36, 70). Resection of the aneurysm and end-to-end anastomosis is another possible treatment (28).

### Endovascular Embolization

With the advances in techniques and materials, endovascular treatment has been an alternative to surgery for the treatment of intracranial pseudoaneurysms. Endovascular procedures include coiling, stent-assisted coiling, occlusion of the parent artery with or without aneurysm, and flow-diversion. The choice of endovascular technique is based on the location of the pseudoaneurysm, vascular anatomy, and clinical status of the patient.

**Figure 2:**
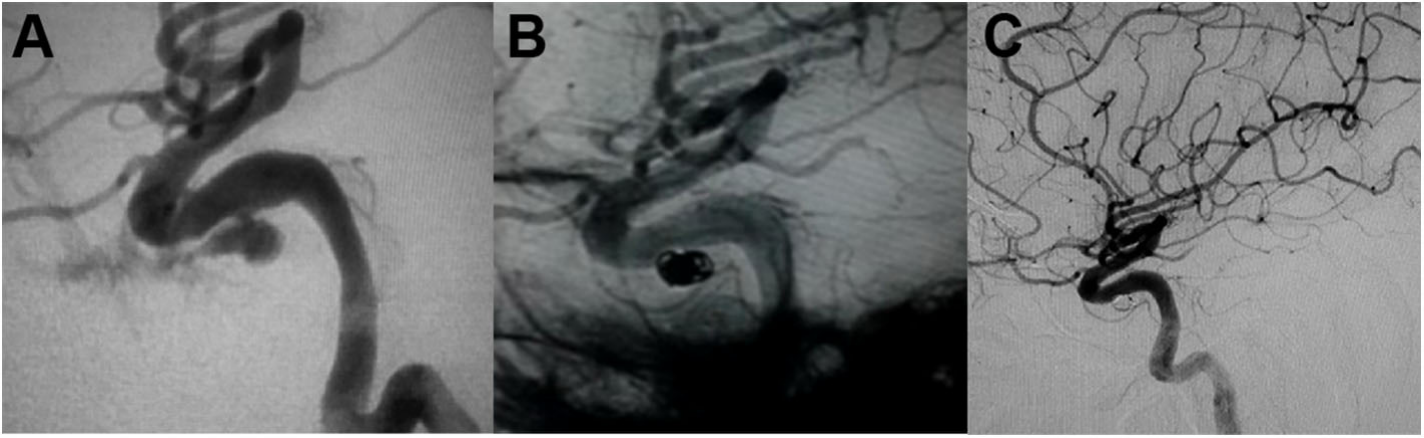
**(A)** Angiography demonstrated the intracavernous iatrogenic pseudoaneurysm of the left internal carotid artery (ICA). **(B)** The pseudoaneurysm was treated by endovascular coiling. **(C)** Angiogram at 4-month follow-up showed no evidence of aneurysmal filling [adapted from Lin et al. (72)].

Packing of the pseudoaneurysm with coils is available for those cases with a narrow neck pseudoaneurysm (52, 71). Because of the fragility of the pseudoaneurysm wall, it has the risk of microcatheter or coil perforation during the procedure. The advantage of selective pseudoaneurysm embolization is the preservation of the parent artery (Figure 2). However, pseudoaneurysm recurrence is still a major issue for patients treated with simple coiling (7, 9, 11, 14, 16, 26, 30, 53, 60, 63). Due to coil impaction into the thrombus (14), flow pulsatility may force into the interstices of the coil mass and lead to recurrent bleeding (27).

**Figure 3:**
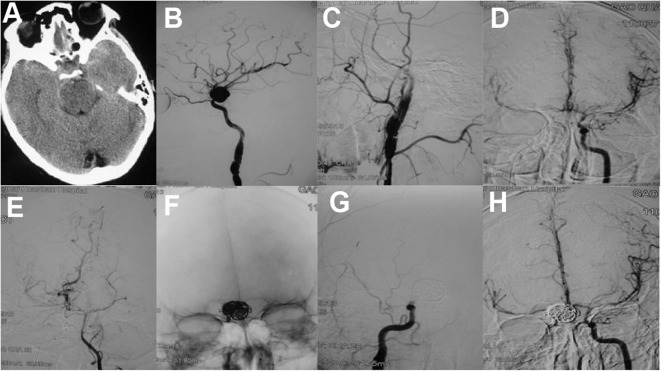
**(A)** An axial CT scan showed skull bone fracture and traumatic subarachonoid hemorrhage. **(B)** Two weeks later, the patient suffered rehemorrhage. A lateral cerebral angiogram of the right ICA demonstrated a large pseudoaneurysm at the C6 segment and a dissection of the C1 segment. Angiogram after balloon occlusion test **(C)** showed good compensation from the anterior communicating artery **(D)** and posterior communicating artery **(E,F)** The pseudoaneurysm and parent artery were trapped with six detachable coils. Postoperative right **(G)** and left **(H)** carotid angiograms showed exclusion of the pseudoaneurysm from the circulation [adapted from Lin et al. (72)].

Therefore, some studies suggested that occlusion of the parent artery and pseudoaneurysm may be the preferred therapy for distal pseudoaneurysms (12, 73). Because of having good collateral supply or retrograde flow from the distal to the trapped segment, occlusion of the parent artery may be safe in distal ACA (24, 68, 73). Coils (39, 73), or liquid embolization agents including glubran (22), n-butylcyanoacrylate (29, 68), and Onyx (12, 24, 74) can be used in parent artery occlusion. Onyx treatment is especially suitable for pseudoaneurysms with minuscule vessel wall as to avoid coiling. However, occlusion of the parent artery is not recommended for pseudoaneurysms of the ICA. Although having negative balloon occlusion test, 22% of patients develop ischemic complications following parent artery occlusion (Figure 3) (16, 52, 75).

Stent-assisted coiling is a promising treatment option for wide-necked pseudoaneurysms (56–58, 65). It allows for the preservation of the parent artery and avoidance of bypass surgery. However, aneurysm recanalization is not uncommon after single stent-assisted coiling (13, 18, 76). Stent-assisted coil embolization followed by stent-within-a-stent technique has been reported as an effective treatment for pseudoaneurysms (58). Overlapping stents with coils effectively prevent rebleeding and regrowth of the pseudoaneurysm. The overlapping stents may divert the flow away from the pseudoaneurysm, accelerate intraaneurysmal thrombosis, and reconstruct the parent artery by promoting neointima formation along the stent (31, 52, 58, 77).

**Figure 4:**
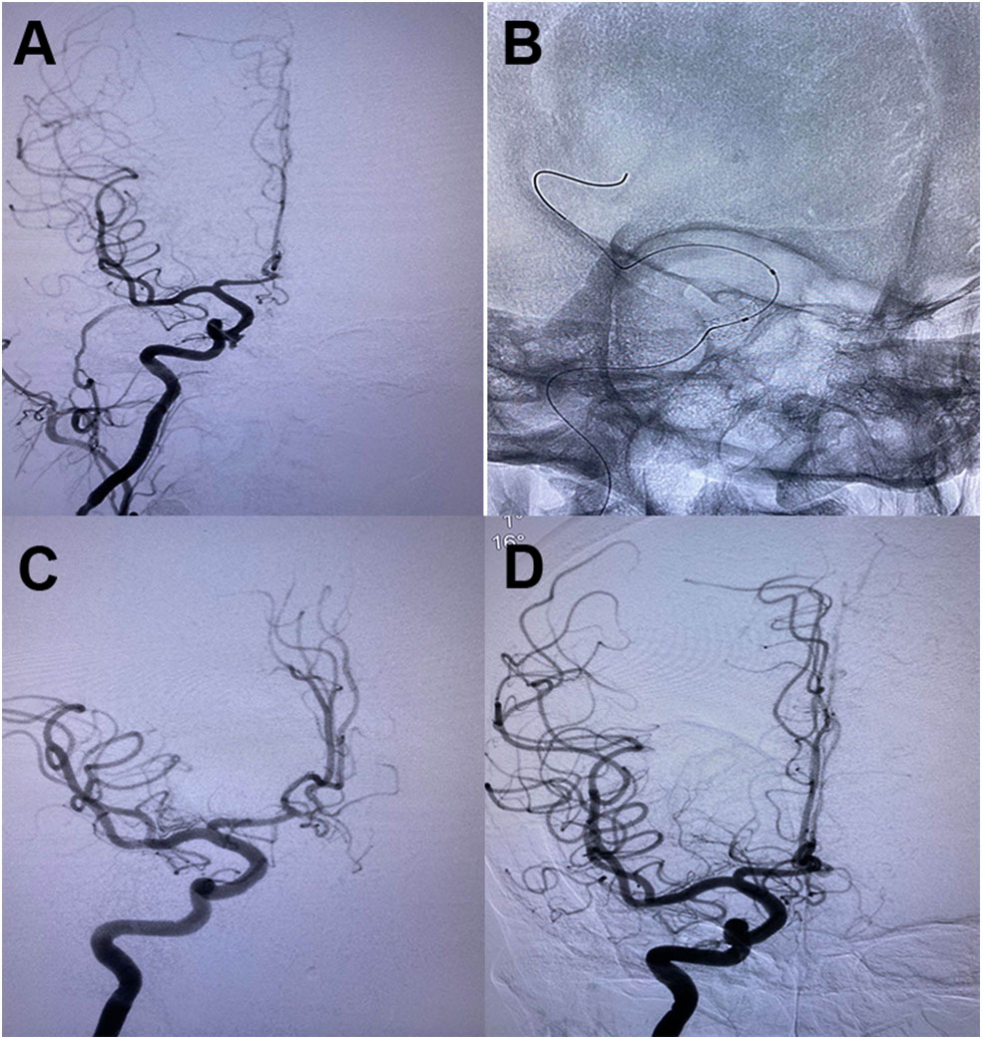
**(A)** Oblique cerebral angiogram showed a pseudoaneurysm in the cavernous segment of righ ICA following endoscopic transsphenoid surgery. **(B)** A 4*13 mm Willis covered stent was deployed across the pseudoaneurysm. **(C)** The control angiogram demonstrated complete obliteration of the pseudoaneurysm with preservation of carotid artery patency. **(D)** A follow-up angiogram showed no recanalization of the aneurysm and patentcy of the parent artery.

Another reconstructive endovascular treatment modality is covered stent implantation (2, 59, 78). Endovascular deployment of covered stents can exclude blood flow through the stent as a physical barrier and keep the normal anatomic flow through the parent artery (Figure 4). Compared with the uncovered stents, covered stents decrease the incidence rate of neointimal proliferation and restenosis, at the same time, decrease embolization risk caused by thrombus debris during the process of stent deployment. However, the flexibility of covered stents and the stiffness of the delivery system are the main limitations for its usage in the tortuous ICA, which may result in dissection and vasospasm. Moreover, the occlusion of important perforators by the covered stent may occur when the pseudoaneurysm originates too close to the origin of the ophthalmic artery, the posterior communicating artery, or the anterior choroidal artery.

Recently, a flow-diverting strategy has been shown to be a promising treatment modality for patients with intracranial pseudoaneurysm. Flow-diverting stent reduces blood flow into the aneurysm, thus promoting thrombosis. It also provides a scaffold for endothelialization and reconstruction of the vessel wall. Previous studies have shown that pseudoaneurysms treated with flow-diverting stents have high complete occlusion rates and low complication rates (6, 7, 18, 19, 26, 30, 53–55, 67, 69, 79). However, the main limitation of flow-diverting stents is delayed aneurysm obliteration due to a lack of immediate thrombosis. It may take weeks for complete aneurysm occlusion, which leaves patients at risk for rebleeding during this time (6, 7, 19, 79). In addition, dural antiplatelet therapy after flow-diverting stent placement may increase the risk of postoperative intracranial hematomas. It should be used judiciously in the setting of ruptured pseudoaneurysms.

### Conservative Treatment

Although high mortality rates of pseudoaneurysms were reported, these data are based on a review of the literature and a collection of only case reports. There is no large sample of pseudoaneurysms in single or multiple centers. Therefore, the true natural history of these pseudoaneurysms is unclear. Complete spontaneous resolution of pseudoaneurysms is considered to be an uncommon occurrence. Previous studies have demonstrated existence of spontaneous resolution in peripheral vessels or intracranial vessels, including the middle meningeal artery (80, 81), basilar artery (82–84), posterior cerebral artery (85) and pericallosal artery (39). The mechanism of spontaneous occlusion remains unclear. It may be due to vascular remodeling response to injury as well as spontaneous thrombus formation (84). Those studies suggest that some specific pseudoaneurysms may at least have a potentially benign course with conservative treatment. Observation might be considered in pseudoaneurysms with decreased size and flow in repeated conventional angiography compared with the initial images (84). However, a recent study reported an unusual course of a cerebral pseudoaneurysm (69). The pseudoaneurysm completely disappeared on the second angiogram, but was found to be enlarged on the third angiogram. Therefore, adequate follow-up is mandatory when conservation treatment is considered or even when the lesions have spontaneous obliteration.

## An Illustrative Case

We present a case of 45-year-old man harboring an invasive pituitary adenoma, in whom an intracavernous carotid artery tear was caused by aggressive curettage of the left cavernous sinus portion of the lesion. Massive intraoperative bleeding was stopped by surgical packing. Subsequent emergent angiography demonstrated an elliptical shaped pseudoaneurysm located in the intracavernous portion of the left ICA (Figure 2A). The pseudoaneurysm was treated by endovascular coiling (Figure 2B). Complete occlusion of the pseudoaneurysm from the circulation with preservation of the parent artery was achieved with placement of six coils. Angiogram at 4-month follow-up showed no evidence of aneurysmal filling (Figure 2C).

## Conclusion

Intracranial pseudoaneurysms are rare pathological entities, representing 1% of all intracranial aneurysms. Rupture of pseudoaneurysm is associated with high rates of morbidity and mortality. Early diagnosis and therapy is critical in those patients with clinical suspicion of a pseudoaneurysm, such as unexplained hemorrhages and epistaxis following a history of head trauma, surgery, or septicemia. Due to spontaneous occlusion of pseudoaneurysms occurred in a few patients, repeated imaging and prompt treatment should be necessary. Endovascular treatments, including coiling with or without stent, covered stent, flow diverting stent, and trapping, should be individualized to aneurysmal location, clinical condition, vascular anatomy, and assessment of the collateral circulation. Microsurgery may be a suitable alternative in cases not amenable to endovascular treatment, yielding favorable outcomes, especially in distal aneurysms or patients with huge hematoma.

## Author Contributions

FX and JS: conception and design. YZ and FX drafted the article. ZL: data collection. All authors listed have made a substantial, direct and intellectual contribution to the work, and approved it for publication.

## Conflict of Interest

The authors declare that the research was conducted in the absence of any commercial or financial relationships that could be construed as a potential conflict of interest.
